# A Junction Temperature Prediction Method Based on Multivariate Linear Regression Using Current Fall Characteristics of SiC MOSFETs

**DOI:** 10.3390/s25154828

**Published:** 2025-08-06

**Authors:** Haihong Qin, Yang Zhang, Yu Zeng, Yuan Kang, Ziyue Zhu, Fan Wu

**Affiliations:** 1 College of Automation Engineering, Nanjing University of Aeronautics and Astronautics, Nanjing 211106, China; yangworkaccount@nuaa.edu.cn (Y.Z.); zhuziyue@nuaa.edu.cn (Z.Z.); wufan198@nuaa.edu.cn (F.W.); 2 School of Electrical Engineering, City University of Hong Kong, Hong Kong, China; zeng_yu@ieee.org; 3 Shaanxi Aviation Electric Co., Ltd., Xi’an 710000, China; kangy031@avic.com

**Keywords:** current fall time, current fall energy loss, junction temperature, condition monitoring, temperature-sensitive electrical parameter (TSEP)

## Abstract

The junction temperature (*T*_j_) is a key parameter reflecting the thermal behavior of Silicon carbide (SiC) MOSFETs and is essential for condition monitoring and reliability assessment in power electronic systems. However, the limited temperature sensitivity of switching characteristics makes it difficult for traditional single temperature-sensitive electrical parameters (TSEPs) to achieve accurate estimation. To address this challenge and enable practical thermal sensing applications, this study proposes an accurate, application-oriented *T*_j_ estimation method based on multivariate linear regression (MLR) using turn-off current fall time (*t*_fi_) and fall loss (*E*_fi_) as complementary TSEPs. First, the feasibility of using current fall time and current fall energy loss as TSEPs is demonstrated. Then, a coupled junction temperature prediction model is developed based on multivariate linear regression using *t*_fi_ and *E*_fi_. The proposed method is experimentally validated through comparative analysis. Experimental results demonstrate that the proposed method achieves high prediction accuracy, highlighting its effectiveness and superiority in MLR approach based on the current fall phase characteristics of SiC MOSFETs. This method offers promising prospects for enhancing the condition monitoring, reliability assessment, and intelligent sensing capabilities of power electronics systems.

## 1. Introduction

Silicon carbide power devices offer advantages such as high-power density [1], excellent high-temperature tolerance, low on-state resistance [2], and high switching frequency [3], making them well-suited for high-power-density power electronics systems. In recent years, they have been increasingly adopted in applications such as rail transportation, aerospace, and wind energy conversion systems [4].

As SiC power device technology continues to mature, reliability concerns in practical applications are becoming increasingly prominent [5]. Under typical operating conditions in power electronics systems, both the absolute junction temperature and its dynamic fluctuations are critical factors influencing device reliability.

**Figure 1:**
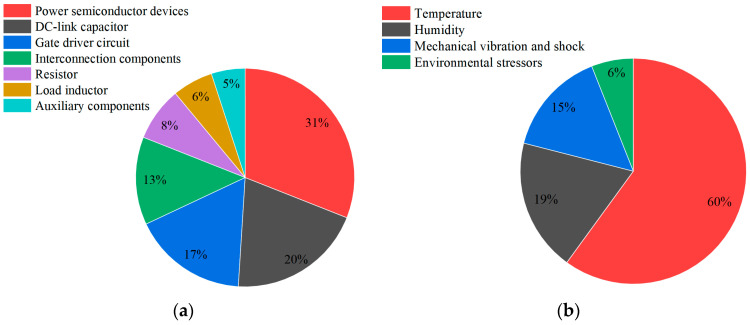
Proportion of failure contributing factors: (**a**) failure rate distribution of power electronics systems; (**b**) failure rate distribution of power devices.

Figure 1 illustrates the distribution of failure modes. Research shows that approximately 31% of power electronics system failures are attributed to power semiconductor device failures, with nearly 60% of these directly linked to thermal factors [6]. Thermal stress (including overheating and temperature fluctuations) is the primary cause of failure in power semiconductor devices, resulting in both transient failures (such as thermal breakdown, bond wire breakage, and solder melting) and aging failures (including bond wire fatigue and solder layer degradation) [7]. Furthermore, the failure rate of power semiconductors approximately doubles for every 10 °C increase in junction temperature [8]. These findings demonstrate the urgent need for effective junction temperature estimation techniques that not only support reliability assessments but also enable integration into practical thermal sensing systems for real-time device health monitoring.

Currently, the methods for measuring junction temperature in power MOSFETs can be broadly categorized into four types: physical contact methods based on sensors, optical methods, thermal resistance network model-based approaches, and temperature-sensitive electrical parameter methods [6]. The physical contact method is invasive and has a slow response time [9,10]; the optical method is limited in practical applications due to its high cost and difficulty in applying it to opaque packaging structures [11,12]. The thermal resistance network model-based approach is a commonly employed method for junction temperature estimation. However, it requires accurate measurement of the module case temperature and the development of a precise thermal resistance network model to be effective [13]. The accuracy of this method largely depends on the accuracy of the thermal impedance network model. Key thermal parameters necessary for constructing a high-precision model are often difficult to obtain, and the model accuracy is easily affected by device aging. Compared with the above three methods, the TSEP method has significant advantages such as fast response speed, high implementation feasibility, and high accuracy [14,15,16]. Commonly used temperature-sensitive electrical parameters (TSEPs) include on-state resistance or voltage drop, switching delay, leakage current, gate threshold voltage, and peak gate current, among others. Real-time tracking of junction temperature is achievable through the measurement of these indicators, and this method has demonstrated promising results, particularly in silicon (Si)-based devices [15,17,18,19].

However, for SiC MOSFETs, certain TSEP-based approaches often fail to deliver optimal monitoring performance and can be problematic for online implementation. First, the inherently high switching frequency of SiC MOSFETs complicates the precise measurement of switching delay indicators; second, due to their excellent high blocking capability, SiC MOSFETs exhibit extremely low leakage current at 25 °C (approximately 1 μA level), making precise measurement difficult [20]; third, although methods based on-state resistance remain applicable, their temperature sensitivity is relatively low (approximately 0.2 mΩ/°C), which limits their ability to meet high-accuracy requirements [21].

N. Baker et al. [22,23] employed the gate peak current as a TSEP, which can be obtained by measuring the voltage drop across the external gate drive resistor. However, this method is susceptible to measurement inaccuracies due to the influence of the gate drive circuit and varying operating conditions. Z. Zhang et al. [24] proposed an online monitoring method for junction temperature prediction based on the turn-off delay time of SiC MOSFETs. F. Yang et al. [25] utilized the turn-on delay time as a temperature-sensitive electrical parameter and further considered the impact of device aging on its effectiveness. However, the switching delay time of SiC MOSFETs exhibits inherently low temperature sensitivity, and, to enhance this sensitivity, both studies [24,25] employed relatively large gate drive resistances, which may in turn affect the device’s switching characteristics. Ying Wang et al. [26] proposed an online junction temperature prediction method based on the falling edge duration of the drain voltage, utilizing auxiliary circuitry to monitor the relevant characteristics of the drain voltage transient. However, this parameter is highly susceptible to measurement errors caused by the parasitic elements of the SiC MOSFET and requires high-precision instrumentation for accurate detection.

Previous studies have demonstrated that the voltage slew rate (d*v*/d*t*) and voltage rise time (*t*_rv_) during the turn-off process are commonly employed for junction temperature estimation in IGBT devices. Lingfeng Shao et al. [27] proposed a hybrid model based on the voltage rise time and voltage rise loss (*E*_rv_) during the IGBT turn-off process. However, the turn-off characteristics are highly susceptible to variations in load conditions, which may compromise the accuracy of the junction temperature prediction model. Moreover, the applicability and temperature sensitivity characteristics of this method for SiC MOSFETs still require further experimental validation.

To address the issue of insufficient temperature sensitivity in the switching characteristics of SiC MOSFETs and the difficulty of accurately predicting junction temperature using traditional single TSEPs, this paper proposes an MLR method for junction temperature estimation based on *t*_fi_ and *E*_fi_. This method is designed not only to improve estimation accuracy but also to enable practical integration into temperature-sensing systems for real-time thermal monitoring of power semiconductor devices. The structure of this paper is as follows: Section 2 first establishes a mathematical model for the current fall stage of SiC MOSFETs, theoretically derives the temperature-sensitive characteristics of *t*_fi_ and *E*_fi_ during the current fall stage, and verifies these characteristics through simulation; Section 3 details the construction method and process of the MLR junction temperature prediction model based on *t*_fi_ and *E*_fi_; Section 4 conducts experimental research using a double-pulse test (DPT) platform, in which both the turn-off characteristics and current fall phase parameters of SiC MOSFETs are measured, and corresponding MLR models are then constructed for comparison and validation. Experimental results demonstrate the accuracy and effectiveness of this junction temperature prediction method; and Section 5 summarizes the key findings and concludes this paper. This work provides a promising foundation for the development of compact, low-cost junction temperature sensors based on SiC MOSFETs.

## 2. Analysis of the Temperature-Dependent Characteristics of Parameters During the Current Fall Phase

### 2.1. Structure and Equivalent Circuit Model of SiC MOSFETs

**Figure 2:**
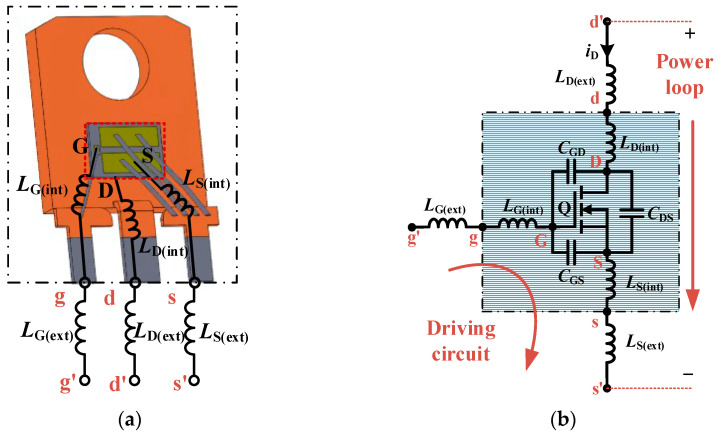
Structure and equivalent circuit of a TO-247-3L SiC MOSFET: (**a**) Schematic of internal package structure. (**b**) Equivalent circuit.

Taking a SiC MOSFET packaged in a TO-247-3L package as an example, the internal structure diagram and the corresponding equivalent circuit of the discrete device are presented in Figure 2. As shown in Figure 2a, the TO-247-3L is one of the most commonly used discrete packaging structures for SiC MOSFETs. In this package, the drain terminal on the backside of the chip is connected to the lead frame through a solder layer, while the gate and source terminals on the front side are connected to their respective leads via aluminum bond wires. The entire assembly is then encapsulated in a plastic molding compound. However, due to the use of metal bond wires and a planar chip layout, the current path from drain to source is relatively long. As a result, the parasitic inductances associated with the internal package and external leads become non-negligible and can significantly influence the device’s high-speed switching performance.

Figure 2b illustrates the equivalent circuit diagram corresponding to the parasitic inductance components of the TO-247-3L package shown in Figure 2a. In this diagram, G, D, and S denote the intrinsic gate, drain, and source terminals of the SiC MOSFET die. The nodes labeled g, d, and s represent the gate, drain, and source terminals after accounting for the internal bonding wires. The nodes g′, d′, and s′ further represent the respective terminals after incorporating both the internal bonding wire inductance and the parasitic inductance introduced by the external package leads. *L*_G(int)_, *L*_D(int)_, and *L*_S(int)_ represent the equivalent parasitic inductances of the internal bonding wires within the device package, while *L*_G(ext)_, *L*_D(ext)_, and *L*_S(ext)_ denote the equivalent parasitic inductances associated with the external package leads and PCB trace connections.

**Figure 3:**
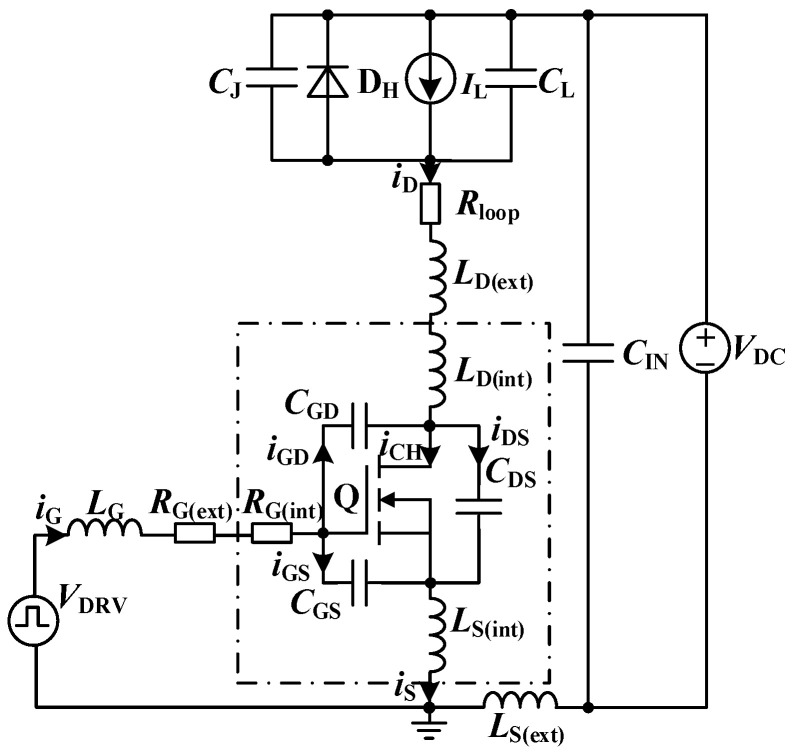
Equivalent circuit of the double-pulse test.

Figure 3 presents the double-pulse test circuit for SiC MOSFETs, incorporating the key parasitic parameters. In Figure 3, *V*_DC_ represents an ideal voltage source equivalent to the DC bus; *I*_L_ denotes an ideal current source equivalent to the load inductance; *C*_L_ accounts for the parasitic capacitance associated with the load inductance; D_H_ is an ideal SiC Schottky Barrier Diode (SBD); *C*_J_ represents the equivalent junction capacitance of the SiC SBD; *C*_GS_, *C*_GD_, and *C*_DS_ represent the gate-to-source, gate-to-drain, and drain-to-source capacitances of the SiC MOSFET, respectively; *L*_D(int)_ and *L*_S(int)_ denote the parasitic inductances associated with the drain and source terminals introduced by the device packaging; *R*_G(int)_ is the intrinsic gate resistance of the SiC MOSFET; *R*_G(ext)_ represents the external gate drive resistance; *L*_G_ represents the parasitic inductance of the gate loop; *L*_D(ext)_ and *R*_loop_ denote the equivalent parasitic inductance and stray resistance, respectively, in the conduction path from the positive terminal of the DC bus to the drain terminal of the SiC MOSFET; and *L*_S(ext)_ corresponds to the parasitic inductance in the return path from the source terminal of the SiC MOSFET to ground.

Figure 3 provides a detailed depiction of the parasitic parameters associated with each component, along with clearly defined current flow directions. Here, *i*_D_ denotes the drain current, *i*_G_ represents the gate current, and *i*_GS_, *i*_GD_, and *i*_DS_ represent the displacement currents through *C*_GS_, *C*_GD_, and *C*_DS_, respectively. *i*_CH_ refers to the channel current of the SiC MOSFET, and *I*_L_ is the load current.

### 2.2. Modeling of the Drain Current Fall Phase

**Figure 4:**
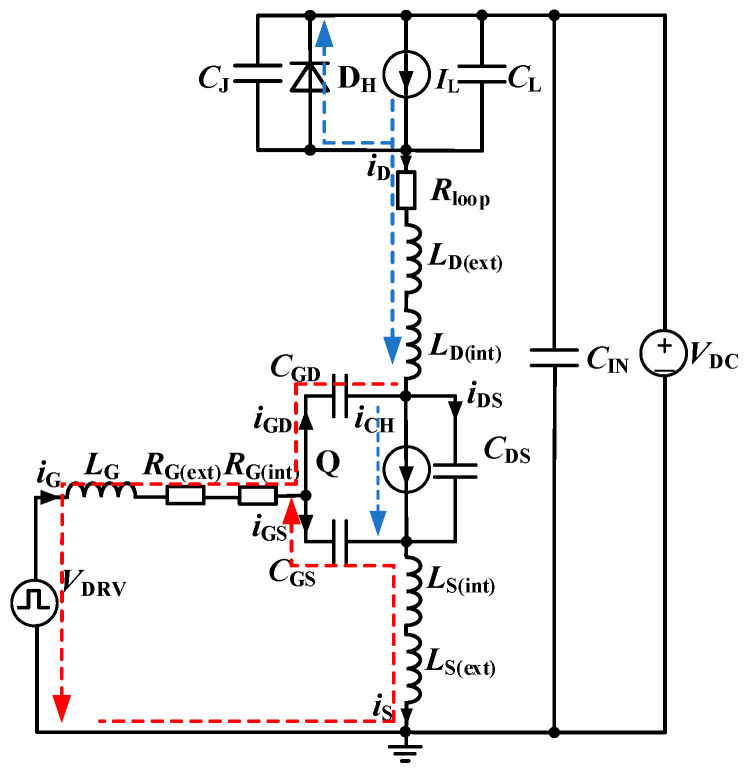
Equivalent circuit during the current fall period.

**Figure 5:**
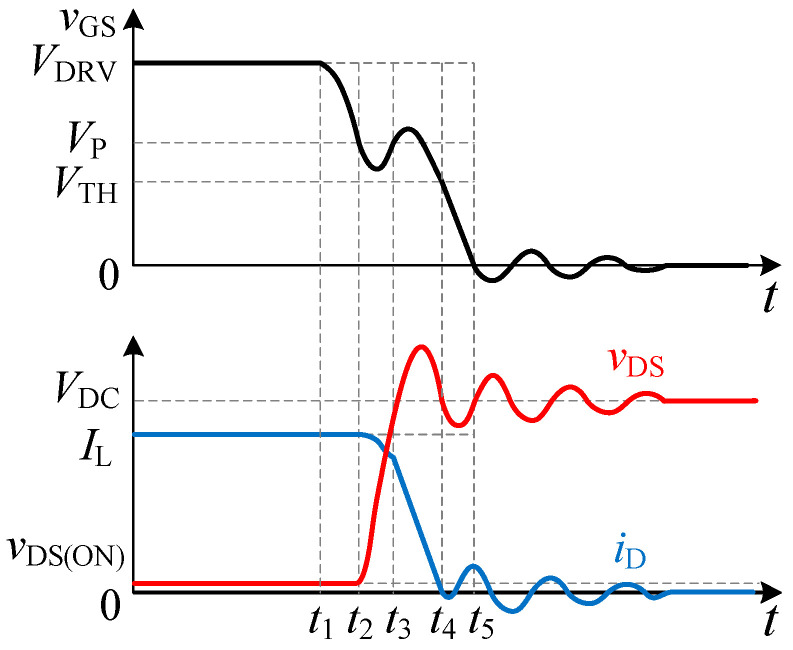
Waveforms of the SiC MOSFET turn-off process.

Figure 4 presents the equivalent circuit for the drain current fall phase (*t*_3_–*t*_4_) under double-pulse testing. The corresponding experimental turn-off waveforms of the SiC MOSFET are depicted in Figure 5. At time *t*_3_, the drain voltage clamps to the bus voltage, preventing further rise. Consequently, the gate-source voltage begins to decrease. With the MOSFET operating in the saturation region, the drain current commences its decay. Simultaneously, the load current commences commutation to the freewheeling diode until *i*_D_ reaches zero. Throughout this interval, *v*_GS_ continues to fall towards the threshold voltage *V*_TH_, governed by the expression:

(1) $$v_{\mathrm{GS}}=V_{\mathrm{DRV}}-R_{G}i_{G}-L_{G}\frac{di_{G}}{dt}-L_{S}\left(\frac{di_{D}}{dt}+\frac{di_{G}}{dt}\right)$$

(2) $$i_{G}=C_{\mathrm{GS}}\frac{dv_{\mathrm{GS}}}{dt}+C_{\mathrm{GD}}\left(\frac{dv_{\mathrm{GS}}}{dt}-\frac{dv_{\mathrm{DS}}}{dt}\right)$$

Since the gate current *i*_G_ is significantly smaller than the drain current *i*_D_, it is neglected in the derivation of the circuit equations.

(3) $$v_{\mathrm{GS}}=V_{\mathrm{DRV}}-R_{G}i_{G}-L_{S}\frac{di_{D}}{dt}$$

By combining the above equations, the governing equation for this stage is obtained:

(4) $$R_{G}C_{\mathrm{ISS}}\frac{dv_{\mathrm{GS}}}{dt}=R_{G}C_{\mathrm{RSS}}\frac{dv_{\mathrm{DS}}}{dt}-v_{\mathrm{GS}}-L_{S}\frac{di_{D}}{dt}+V_{\mathrm{DRV}}$$

(5) $$v_{\mathrm{DS}}=V_{\mathrm{DC}}-L_{\mathrm{loop}}\frac{di_{D}}{dt}-R_{\mathrm{loop}}i_{D}$$

(6) $$i_{D}=g_{\mathrm{fs}}\cdot \left(v_{\mathrm{GS}}-V_{\mathrm{TH}}\right)+C_{\mathrm{GD}}\frac{dV_{\mathrm{GD}}}{dt}+C_{\mathrm{DS}}\frac{dV_{\mathrm{DS}}}{dt}$$

The expression for the drain current fall rate is derived as follows:

(7) $$\frac{di_{D}}{dt}=-\frac{g_{\mathrm{fs}}(V_{p}-V_{\mathrm{EE}})}{R_{G}(C_{\mathrm{GS}}+C_{\mathrm{GD}})+g_{\mathrm{fs}}L_{S}}$$

The expression for the drain current *i*_D_ during the interval *t*_3_ to *t*_4_ is derived as follows:

(8) $$i_{D}(t)=I_{L}-\frac{g_{\mathrm{fs}}(V_{p}-V_{\mathrm{EE}})}{R_{G}(C_{\mathrm{GS}}+C_{\mathrm{GD}})+g_{\mathrm{fs}}L_{S}}(t_{4}-t_{3})$$

The duration of this interval is then calculated as follows:

(9) $$t_{\mathrm{fi}}=t_{4}-t_{3}=I_{L}\frac{R_{G}(C_{\mathrm{GS}}+C_{\mathrm{GD}})+g_{\mathrm{fs}}L_{S}}{g_{\mathrm{fs}}(V_{p}-V_{\mathrm{EE}})}$$

The power dissipation during this switching phase is given by the following:

(10) $$E_{\mathrm{fi}}=\int_{t_{3}}^{t_{4}}\left[v_{\mathrm{DS}}\left(t\right)\cdot i_{D}\left(t\right)\right]dt=\frac{1}{2}V_{\mathrm{DC}}\cdot I_{L}\cdot (R_{G}C_{\mathrm{ISS}}+g_{\mathrm{fs}}L_{S})\cdot \ln\frac{V_{\mathrm{DRV}}+V_{\mathrm{TH}}}{V_{\mathrm{TH}}}$$

### 2.3. Temperature-Dependent Characteristics Analysis of t_fi_ and E_fi_ During the Current Fall Phase

**Figure 6:**
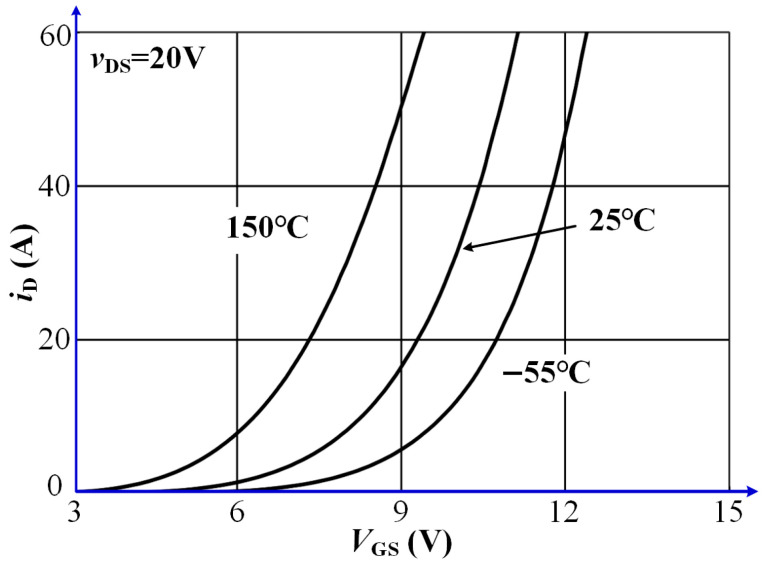
Transfer characteristics of the NTHL020N090SC1 SiC MOSFET.

The transfer characteristics of a SiC MOSFET describe the relationship between the drain current and the gate-source voltage. This relationship is typically represented by a transfer characteristic curve, whose slope corresponds to the device’s transconductance. A steeper slope indicates higher transconductance, reflecting greater sensitivity of the drain current to changes in gate voltage. Figure 6 presents the transfer characteristic curves of the SiC MOSFET model NTHL020N090SC1 from the ON Semiconductor. Within a certain range, the slope of the curve increases with the gate-source voltage, indicating enhanced transconductance. A comparison of the curves at different temperatures reveals that, with rising temperature, the entire transfer curve shifts upward, and the threshold voltage decreases progressively. This indicates that higher temperatures facilitate device turn-on, as a lower gate voltage is required to achieve the same drain current.

The transfer characteristic curve illustrates the relationship between the gate-source voltage *v*_GS_ and the drain current *i*_D_ under a constant drain-source voltage. The slope at each point on the curve reflects the device’s transconductance, which characterizes the amplification capability of the MOSFET. Among these parameters, the gate threshold voltage and transconductance are particularly significant in the output characteristics of SiC MOSFETs, both exhibiting strong temperature dependence. The threshold voltage can be expressed by Equation (11):

(11) $$V_{\mathrm{TH}}=\frac{\sqrt{4\varepsilon_{s}kTN_{A}ln(N_{A}/n_{i})}}{C_{\mathrm{ox}}}+\frac{2kT}{q}ln\left(\frac{N_{A}}{n_{i}}\right)$$

In this equation, *n*_i_ denotes the intrinsic carrier concentration, while *n* and *p* represent the concentrations of electrons and holes, respectively. *k* is the Boltzmann constant, *T* is the absolute temperature in kelvins, *N*_A_ is the acceptor doping concentration, *ε*_s_ is the relative permittivity of the semiconductor, *C*_OX_ is the characteristic capacitance of the oxide layer, and *q* is the unit charge. The intrinsic carrier concentration of SiC increases with temperature, and this rise predominantly influences the variation in the threshold voltage. As a result, the threshold voltage of SiC MOSFETs exhibits a negative temperature coefficient, decreasing as the temperature increases.

In the transfer characteristic curve of a MOSFET, the transconductance represents the ratio of the change in drain current *i*_D_ to the change in gate-source voltage *v*_GS_, reflecting the degree to which the gate-source voltage controls the drain current. It can be mathematically expressed as shown in Equation (12):

(12) $$g_{\mathrm{fs}}=\frac{Z\mu_{\mathrm{ni}}C_{\mathrm{OX}}}{L_{\mathrm{CH}}}(v_{\mathrm{GS}}-V_{\mathrm{TH}})$$

In this equation, *Z* represents the channel width, *μ*_ni_ denotes the electron mobility in the inversion channel, *v*_GS_ is the gate-to-source voltage, and *L*_CH_ represents the channel length. The electron mobility in the inverted channel, *μ*_ni_, can be expressed as a combination of several components derived from typical scattering mechanisms, including bulk lattice scattering (*μ*_B_), surface roughness scattering (*μ*_sr_), surface phonon scattering (*μ*_ph_), and interface state scattering (*μ*_it_):

(13) $$\mu_{\mathrm{ni}}^{-1}=\mu_{B}^{-1}+\mu_{\mathrm{it}}^{-1}+\mu_{\mathrm{ph}}^{-1}+\mu_{\mathrm{sr}}^{-1}$$

The inversion channel electron mobility of SiC MOSFETs increases with temperature up to approximately 600 K [28]. On the other hand, the threshold voltage of SiC MOSFETs decreases with rising temperature, so the transconductance of SiC MOSFETs increases with rising temperature.

Based on the analysis and modeling of the current fall phase, the duration of this phase can be expressed as follows:

(14) $$t_{\mathrm{fi}}=I_{L}\frac{R_{G}(C_{\mathrm{GS}}+C_{\mathrm{GD}})+g_{\mathrm{fs}}L_{S}}{g_{\mathrm{fs}}(V_{p}-V_{\mathrm{EE}})}$$

By differentiating the current fall time with respect to the junction temperature *T*_j_, the following expression is obtained:

(15) $$\frac{dt_{\mathrm{fi}}}{dT_{j}}=\frac{I_{L}g_{\mathrm{fs}}(V_{\mathrm{TH}}-V_{\mathrm{EE}})(\frac{1}{L_{S}g_{\mathrm{fs}}}+R_{G}\frac{\left(C_{\mathrm{GS}}+C_{\mathrm{GD}}\right)}{L_{S}}-1)}{{\left(g_{\mathrm{fs}}V_{\mathrm{TH}}+I_{L}-g_{\mathrm{fs}}V_{\mathrm{EE}}\right)}^{2}}\frac{dg_{\mathrm{fs}}}{dT_{j}}-\frac{I_{L}(R_{G}(C_{\mathrm{GS}}+C_{\mathrm{GD}})+g_{\mathrm{fs}}L_{S})g_{\mathrm{fs}}}{{\left(g_{\mathrm{fs}}V_{\mathrm{TH}}+I_{L}-g_{\mathrm{fs}}V_{\mathrm{EE}}\right)}^{2}}\frac{dV_{\mathrm{TH}}}{dT_{j}}$$

Based on the above analysis, it can be concluded that the transconductance *g*_fs_ exhibits a positive temperature coefficient, whereas the threshold voltage *V*_TH_ exhibits a negative temperature coefficient. Therefore, the following relationship can be derived:

(16) $$\frac{dt_{\mathrm{fi}}}{dT_{j}}>0$$

Therefore, the current fall time during the turn-off process exhibits a positive temperature coefficient, indicating that the current fall duration of the SiC MOSFET increases progressively with rising temperature.

The energy loss during the current fall phase can be expressed as follows:

(17) $$E_{\mathrm{fi}}=\int_{t_{3}}^{t_{4}}\left[v_{\mathrm{DS}}\left(t\right)\cdot i_{D}\left(t\right)\right]dt=\frac{1}{2}V_{\mathrm{DC}}\cdot I_{L}\cdot (R_{G}C_{\mathrm{ISS}}+g_{\mathrm{fs}}L_{S})\cdot \ln\frac{V_{\mathrm{DRV}}+V_{\mathrm{TH}}}{V_{\mathrm{TH}}}$$

Similarly, differentiating the energy loss *E*_fi_ during the current fall phase with respect to the junction temperature *T*_j_ yields the following:

(18) $$\frac{dE_{\mathrm{fi}}}{dT_{j}}=\frac{1}{2}V_{\mathrm{DC}}I_{L}\cdot \left[L_{S}\cdot \frac{dg_{\mathrm{fs}}}{dT_{j}}\cdot \ln\left(\frac{V_{\mathrm{DRV}}+V_{\mathrm{TH}}}{V_{\mathrm{TH}}}\right)-\frac{(R_{G}C_{\mathrm{ISS}}+L_{S}g_{\mathrm{fs}})\cdot V_{\mathrm{DRV}}\cdot \frac{dV_{\mathrm{TH}}}{dT_{j}}}{V_{\mathrm{TH}}(V_{\mathrm{DRV}}+V_{\mathrm{TH}})}\right]$$

From the above analysis, the transconductance *g*_fs_ exhibits a positive temperature coefficient, while the threshold voltage *V*_TH_ shows a negative temperature coefficient. As a result, the derivative of *E*_fi_ with respect to junction temperature *T*_j_ is positive, d*E*_fi_/d*T*_j_ > 0.

Therefore, the energy loss during the current fall stage of the turn-off process exhibits a positive temperature coefficient, indicating that the current fall energy loss of the SiC MOSFET increases progressively with rising junction temperature.

From the above analysis, it can be seen that, during the turn-off process, the current fall time and current fall loss both increase with the rise in junction temperature, exhibiting a positive correlation with junction temperature.

### 2.4. Simulation Analysis of Temperature-Dependent Characteristics

Based on the above analysis, it can be concluded that the parameter characteristics during the current fall stage of SiC MOSFETs in practical circuit applications are significantly influenced by the junction temperature and exhibit a monotonic trend with temperature variation. These characteristics can therefore serve as TSEPs for predicting the junction temperature of SiC MOSFETs.

This section presents simulation results conducted using LTspice software XVII. The parasitic inductance parameters of both the power and gate drive circuits are treated as adjustable variables, while all other parameters are set according to actual operating conditions. The simulations aim to verify the impact of varying junction temperatures on the current fall time and current fall loss. The detailed parameters of the SiC MOSFET double-pulse test circuit used in the simulation are summarized in Table 1.

**Figure 7:**
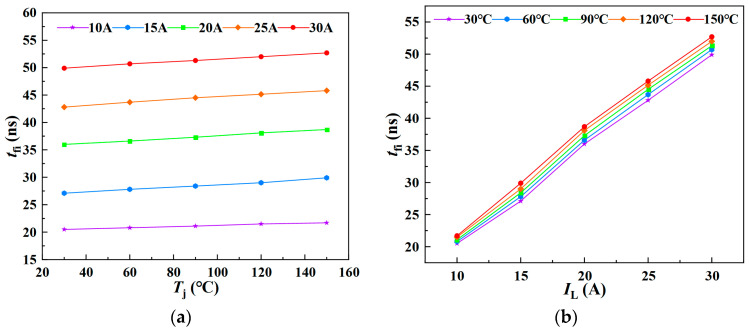
Variation of *t*_fi_ with load current and junction temperature: (**a**) Dependence of *t*_fi_ on junction temperature; (**b**) dependence of *t*_fi_ on load current.

A double-pulse simulation circuit was implemented in LTspice XVII, with the device model’s junction temperature set at five discrete levels: 30 °C, 60 °C, 90 °C, 120 °C, and 150 °C. The current fall time *t*_fi_ and current fall loss *E*_fi_ parameters were extracted and recorded from the simulation results. Figure 7 presents the simulated relationships among junction temperature, load current, and current fall time during the turn-off process of the SiC MOSFET. The results align well with the theoretical analysis, demonstrating that the current fall time increases with both rising junction temperature and load current during turn-off.

**Figure 8:**
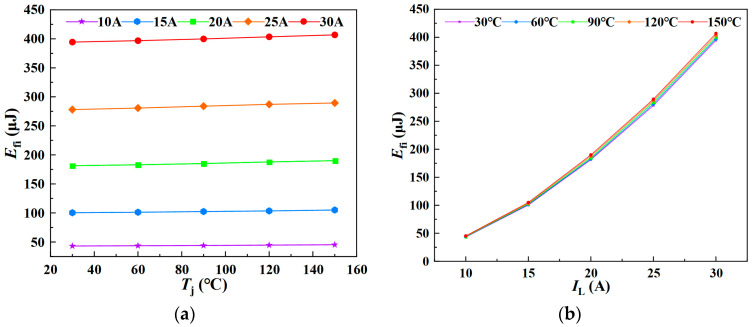
Variation of *E*_fi_ with load current and junction temperature: (**a**) dependence of *E*_fi_ on junction temperature; (**b**) dependence of *E*_fi_ on load current.

Figure 8 illustrates the simulated relationship between junction temperature, load current, and current fall energy loss during the turn-off process. The simulation results are consistent with the theoretical analysis, indicating that the current fall energy loss increases with both higher junction temperatures and greater load currents.

Based on the above simulation analysis, it can be observed that, during the current fall phase of the SiC MOSFET turn-off process, both the current fall time *t*_fi_ and the associated energy loss *E*_fi_ increase with rising junction temperature. The simulation results further demonstrate that *t*_fi_ and *E*_fi_ exhibit a linear correlation with *T*_j_, effectively reflecting the junction temperature variations in the SiC MOSFET.

Based on the aforementioned theoretical and simulation analyses, it can be concluded that both *t*_fi_ and *E*_fi_ exhibit a positive correlation with junction temperature during the turn-off process of the SiC MOSFET. This confirms their feasibility as TSEPs. Accordingly, this paper proposes an MLR approach to estimate the junction temperature based on *t*_fi_ and *E*_fi_.

## 3. Junction Temperature Estimation Method Based on MLR During the Current Fall Phase of SiC MOSFETs

Through experimental testing on a calibration platform, temperature-sensitive electrical parameter data under various operating conditions and junction temperatures can be obtained. By fitting a three-dimensional surface model involving current fall time *t*_fi_, current fall energy loss *E*_fi_, load current *I*_L_, and junction temperature *T*_j_, MLR models for *t*_fi_-*T*_j_-*I*_L_ and *E*_fi_-*T*_j_-*I*_L_ relationships can be established, as expressed in Equations (19) and (20):

(19) $$t_{\mathrm{fi}}=\alpha_{1}T_{j}+\beta_{1}I_{L}+\eta_{1}$$

(20) $$E_{\mathrm{fi}}=\alpha_{2}T_{j}+\beta_{2}I_{L}-\eta_{2}$$

The general form of the multiple linear regression model is defined as shown in Equation (21):

(21) $$Y=\beta_{0}+\beta_{1}X_{1}+\beta_{2}X_{2}+\ldots +\beta_{m}X_{m}$$

This equation defines the structure of a multiple linear regression model. In this context, *Y* denotes the dependent variable, while *X*_1_, *X*_2_, ..., *X*_m_ represent the independent variables. The partial regression coefficients *β*_j_ (j = 1, 2, ..., m) represent the average change in *Y* when *X*_j_ increases or decreases by one unit while keeping the other independent variables constant.

Simulation results demonstrate that the TSEPs increase with rising junction temperature *T*_j_ and load current *I*_L_. In this context, *t*_fi_ and *E*_fi_ serve as dependent variables, while *T*_j_ and *I*_L_ are considered independent variables. Accordingly, a multiple linear regression model was established to describe the relationship between the temperature-sensitive parameters and the junction temperature and load current. However, for the purpose of junction temperature prediction, it is essential to construct a regression model in which *T*_j_ is the dependent variable and the remaining parameters serve as independent variables, as expressed in Equation (22).

(22) $$T_{j}=\alpha I_{L}+\beta t_{\mathrm{fi}}+\eta$$

However, since there is no direct linear causal relationship between *I*_L_ and *T*_j_ in this model, the fitting accuracy is limited, and the explanatory power for the dependent variable is relatively weak. Therefore, it is necessary to eliminate the influence of load current to improve the accuracy of the temperature-sensitive electrical parameter method when applied to junction temperature prediction of SiC MOSFETs. By coupling multiple TSEPs, the effect of load current can be eliminated through matrix operations, enabling the extraction of the junction temperature. The implementation process is as follows:

(23) $$\left[\begin{array}{l} t_{\mathrm{fi}}\\ E_{\mathrm{fi}} \end{array}\right]=\left[\begin{array}{cc} \alpha_{1} & \beta_{1}\\ \alpha_{2} & \beta_{2} \end{array}\right]\left[\begin{array}{l} T_{j}\\ I_{L} \end{array}\right]+\left[\begin{array}{l} \eta_{1}\\ \eta_{2} \end{array}\right]$$

(24) $$\left[\begin{array}{l} T_{j}\\ I_{L} \end{array}\right]={\left[\begin{array}{cc} \alpha_{1} & \beta_{1}\\ \alpha_{2} & \beta_{2} \end{array}\right]}^{-1}\left[\begin{array}{l} t_{\mathrm{fi}}\\ E_{\mathrm{fi}} \end{array}\right]+\left[\begin{array}{c} \eta_{3}\\ \eta_{4} \end{array}\right]$$

Then, the following expression can be obtained:

(25) $$T_{j}=\alpha_{3}t_{\mathrm{fi}}-\beta_{3}E_{\mathrm{fi}}-\eta_{3}$$

An MLR model for predicting the junction temperature of *T*_j_-*t*_fi_-*E*_fi_ was established based on the three-dimensional data of *t*_fi_-*T*_j_-*I*_L_ and *E*_fi_-*T*_j_-*I*_L_. By incorporating multiple TSEPs, the influence of load current *I*_L_ was effectively decoupled, thereby enhancing the accuracy of junction temperature estimation for SiC MOSFETs using electrical parameter-based methods.

**Figure 9:**
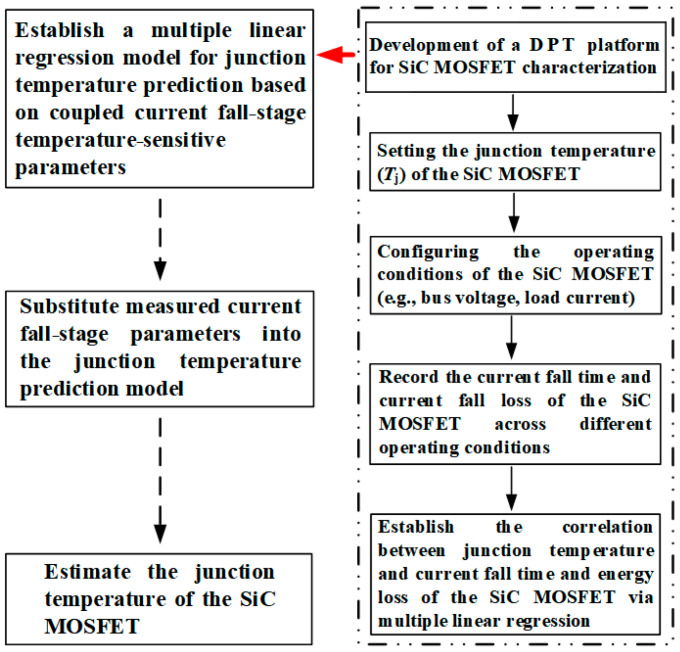
Workflow for developing MLR junction temperature prediction model.

Therefore, an MLR method for junction temperature prediction based on the current fall characteristics of SiC MOSFETs is proposed, as illustrated in Figure 9. Initially, a DPT platform was established to facilitate model construction. To ensure that the parasitic parameters in the calibration setup closely match those in the actual operating circuit, both circuits should be designed with similar structural configurations and PCB layouts.

Experimental validation was performed using a DPT platform, with an oscilloscope employed to capture the temperature-dependent characteristics of the current fall phase during the turn-off process. The operating conditions for the SiC MOSFET were set, including the junction temperature *T*_j_, DC bus voltage, and load current. The junction temperature was controlled via an intelligent temperature regulation unit. The *t*_fi_ and *E*_fi_ of the SiC MOSFET during the turn-off process were measured under various operating conditions and different junction temperatures. The relationship between junction temperature and TSEPs was fitted using MATLAB R2023b or Origin 2021 software. Based on the coupling of multiple TSEPs in the current fall phase, an MLR model was established to predict the junction temperature of the SiC MOSFET. Finally, by substituting the measured values of *t*_fi_ and *E*_fi_ into the established *T*_j_-*t*_fi_-*E*_fi_ junction temperature prediction model, the junction temperature *T*_j_ of the SiC MOSFET can be accurately estimated. This implementation not only enhances temperature estimation accuracy but also provides a practical foundation for developing compact, real-time junction temperature sensing solutions in SiC-based power electronic systems.

## 4. Experimental Validation

### 4.1. Experimental Platform

**Figure 10:**
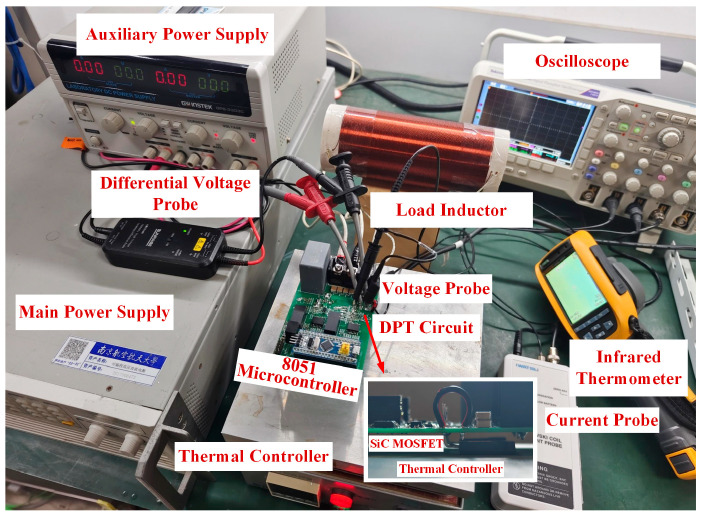
Experimental DPT platform for SiC MOSFET characterization.

Figure 10 illustrates the DPT platform developed in this study for characterizing SiC MOSFETs. The main components of the platform include the following: (1) an intelligent temperature control chamber; (2) the DPT circuit; (3) a high-power DC power supply; (4) an infrared thermometer; (5) auxiliary power supplies; (6) high-voltage differential probes and high-bandwidth current probes; (7) a digital storage oscilloscope; and (8) load inductor.

The NTHL020N090SC1 SiC MOSFET was selected as the device under test (DUT). The DUT was mounted on a temperature-controlled heating stage equipped with an insulation pad. The stage temperature was varied from 30 °C to 150 °C in 20 °C increments (30 °C, 50 °C, 70 °C, 90 °C, 110 °C, 130 °C, 150 °C). At each temperature, the DUT was allowed to thermally stabilize for 5 min prior to measurement. Switching characteristics were then measured at load currents ranging from 10 A to 30 A in 5 A steps.

A constant-temperature heating duration of 5 min ensures sufficient thermal conduction within the chip, allowing the case temperature (*T*_c_) and junction temperature *T*_j_ of the device to closely approximate the set temperature of the heating platform. Furthermore, to minimize the influence of self-heating during the calibration process, each pulse signal is limited to only a few microseconds. The heat generated by the chip during this extremely short period is negligible and does not significantly affect the junction temperature. Once the MOSFET chip reaches the target temperature, the corresponding voltage and current waveforms are captured using a digital oscilloscope, and the measurement data are subsequently recorded for analysis.

In addition, the platform adopts the same gate driver circuit design as that used in actual power electronic converters and allows configurable experimental conditions to emulate real operating points. This ensures that the switching characteristics obtained from experiments essentially reflect the behavior of the device under test within practical converters under identical driving conditions, thereby maximizing the comparability of the extracted parameters and the relevance of the model calibration data. This experimental consistency also enhances the applicability of the proposed method for integration into embedded temperature-sensing systems, where the extracted TSEPs can serve as internal indicators for real-time junction temperature monitoring without requiring additional temperature sensors or circuitry. Table 2 presents the operating conditions of the SiC MOSFET double-pulse test platform.

### 4.2. Experimental Results and Analysis

**Figure 11:**
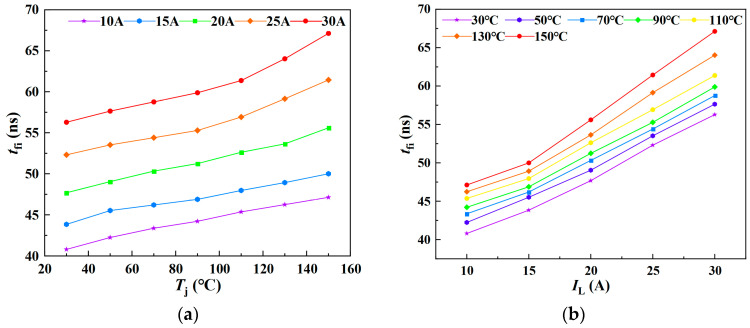
Current fall time under varying junction temperatures and load currents: (**a**) Junction temperature dependence of current fall time. (**b**) Load current dependence of current fall time.

As illustrated in Figure 11a,b, the variation of *t*_fi_ with junction temperature under different load currents and its variation with load current at different junction temperatures are depicted, respectively. The experimental results demonstrate that the current fall time during the turn-off phase increases approximately linearly with both rising junction temperature and load current. Based on these observations, a multiple linear regression model for *t*_fi_-*T*_j_-*I*_L_ can be established.

**Figure 12:**
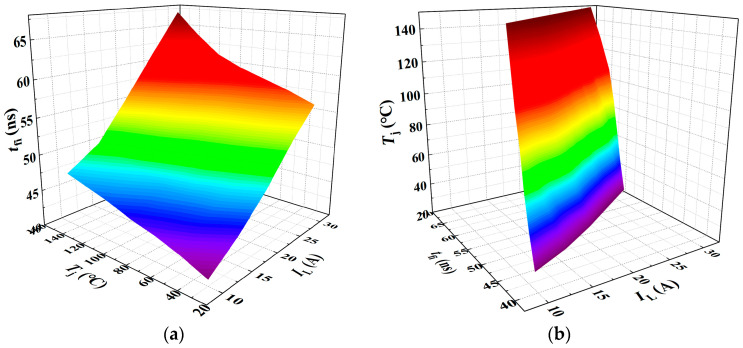
A 3D parametric model of *t*_fi_ = *f*(*T*_j_, *I*_L_): (**a**) *t*_fi_ as dependent variable in multivariate analysis; (**b**) *T*_j_ as dependent variable in multivariate analysis. The color gradient in the 3D plot primarily reflects variations in the vertical-axis amplitude, functioning as a contour-like representation. The transition from purple to red corresponds to changes in the amplitude value. This chromatic encoding visually enhances the interpretability of how the dependent variable evolves with the independent variables within the three-dimensional space.

As shown in Figure 12, a three-dimensional surface model of *t*_fi_-*T*_j_-*I*_L_ is constructed and fitted using Matlab R2023b/Origin 2021 software based on multiple linear regression.

When *t*_fi_ is treated as the dependent variable, the resulting regression model is expressed in Equation (26), with a goodness of fit *R*^2^ of 98.08%. Conversely, when *T*_j_ is taken as the dependent variable, the corresponding regression model is given in Equation (27), with an *R*^2^ value of 88.88%.

(26) $$t_{\mathrm{fi}}=0.06439T_{j}+0.84329I_{L}+29.25011$$

(27) $$T_{j}=-11.72593I_{L}+13.90505t_{\mathrm{fi}}-397.30461$$

**Figure 13:**
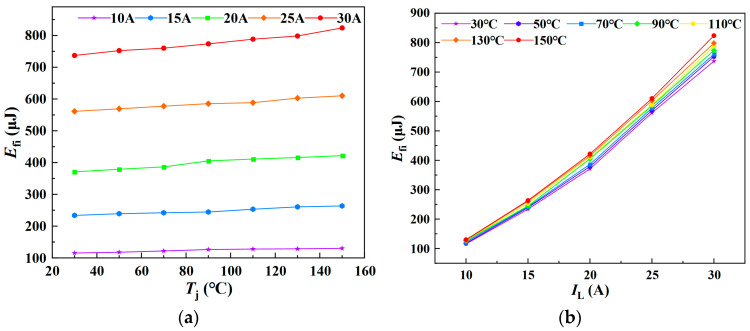
Current fall loss under varying junction temperatures and load currents: (**a**) Junction temperature dependence of current fall loss. (**b**) Load current dependence of current fall loss.

Similarly, *E*_fi_ was measured under varying junction temperatures and load currents, with the experimental results presented in Figure 13. Figure 13a,b illustrate the variation of *E*_fi_ with junction temperature at different load currents, and with load current at different junction temperatures, respectively. The results indicate that the current fall loss during the turn-off phase increases approximately linearly with both junction temperature and load current. Accordingly, a multiple linear regression model for *E*_fi_ as a function of *T*_j_ and *I*_L_ was established.

**Figure 14:**
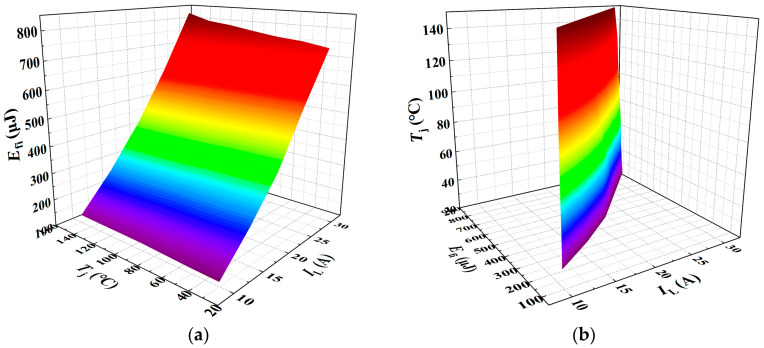
A 3D parametric model of *E*_fi_ = *f*(*T*_j_, *I*_L_): (**a**) *E*_fi_ as dependent variable in multivariate analysis. (**b**) *T*_j_ as dependent variable in multivariate analysis. The color gradient in the 3D plot primarily reflects variations in the vertical-axis amplitude, functioning as a contour-like representation. The transition from purple to red corresponds to changes in the amplitude value. This chromatic encoding visually enhances the interpretability of how the dependent variable evolves with the independent variables within the three-dimensional space.

As illustrated in Figure 14, the three-dimensional surface model of *E*_fi_ with respect to *T*_j_ and *I*_L_ is presented.

A multiple linear regression model was fitted using Matlab R2023b/Origin 2021 software. When *E*_fi_ is treated as the dependent variable, the resulting regression model is given in Equation (28), yielding a goodness of fit *R*^2^ of 99.04%. Conversely, when *T*_j_ is taken as the dependent variable, the corresponding regression model is shown in Equation (29), with a significantly lower goodness of fit *R*^2^ of only 28.91%.

(28) $$E_{\mathrm{fi}}=0.038287T_{j}+32.82411I_{L}-264.59014$$

(29) $$T_{j}=-27.70953I_{L}+0.84418E_{\mathrm{fi}}+284.27306$$

The multiple linear regression models derived from fitting the *t*_fi_-*T*_j_-*I*_L_ and *E*_fi_-*T*_j_-*I*_L_ datasets indicate that both *t*_fi_ and *E*_fi_ increase linearly with rising junction temperature and load current. These models, with *t*_fi_ and *E*_fi_ as dependent variables and *T*_j_ and *I*_L_ as independent variables, demonstrate a high goodness of fit, effectively capturing the temperature- and current-dependent variations in the electrical parameters. However, the fitted MLR models exhibit varying degrees of goodness of fit. The *R*^2^ value for the *T*_j_-*I*_L_-*t*_fi_ three-dimensional model is 88.88%, whereas that for the *T*_j_-*I*_L_-*E*_fi_ model is only 28.91%. Therefore, relying on a single temperature-sensitive electrical parameter to estimate the junction temperature may introduce substantial errors, resulting in reduced prediction accuracy.

Since switching losses in SiC MOSFETs primarily occur during the voltage rise and current fall intervals of the turn-off process, the total energy dissipated in these intervals is defined as the turn-off loss *E*_off_, and the corresponding duration is defined as the turn-off time *t*_off_. In the following sections, three-dimensional models of *t*_off_ and *E*_off_ are established, followed by the development of a junction temperature prediction model. A comparative analysis of their performance is also presented.

**Figure 15:**
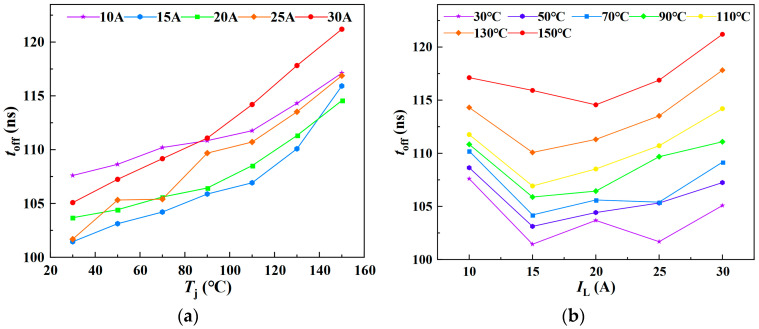
Turn-off time under varying junction temperatures and load currents: (**a**) Junction temperature dependence of turn-off time. (**b**) Load current dependence of turn-off time.

**Figure 16:**
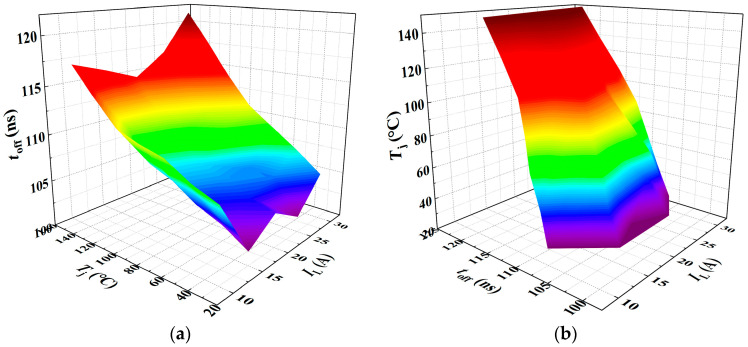
A 3D parametric model of *t*_off_ = *f*(*T*_j_, *I*_L_): (**a**) *t*_off_ as dependent variable in multivariate analysis; (**b**) *T*_j_ as dependent variable in multivariate analysis. The color gradient in the 3D plot primarily reflects variations in the vertical-axis amplitude, functioning as a contour-like representation. The transition from purple to red corresponds to changes in the amplitude value. This chromatic encoding visually enhances the interpretability of how the dependent variable evolves with the independent variables within the three-dimensional space.

As shown in Figure 15a,b, the variations of *t*_off_ with junction temperature under different load currents, and with load current under different junction temperatures, are presented, respectively. The experimental results indicate that the turn-off time increases approximately linearly with increasing junction temperature. Figure 16 presents the three-dimensional surface model of *t*_off_-*T*_j_-*I*_L_. When *t*_off_ is treated as the dependent variable, the corresponding multiple linear regression model is given in Equation (30), yielding a goodness of fit *R*^2^ of 73.85%. Conversely, when *T*_j_ is considered the dependent variable, the fitted model is shown in Equation (31), with an *R*^2^ value of 73.53%.

(30) $$t_{\mathrm{off}}=0.10455T_{j}+0.075I_{L}+98.56246$$

(31) $$T_{j}=-0.53864I_{L}+7.18183t_{\mathrm{off}}-685.43913$$

**Figure 17:**
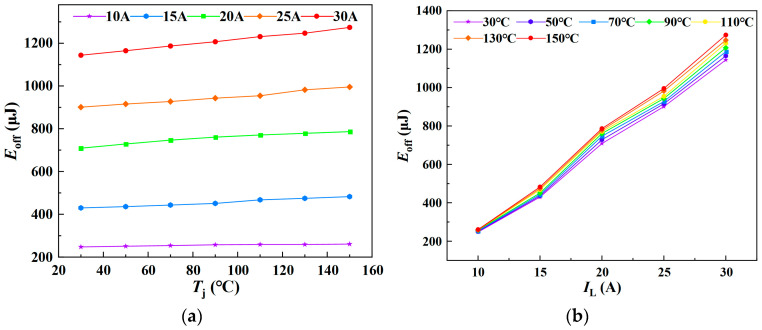
Turn-off loss under varying junction temperatures and load currents: (**a**) Junction temperature dependence of turn-off loss. (**b**) Load current dependence of turn-off loss.

**Figure 18:**
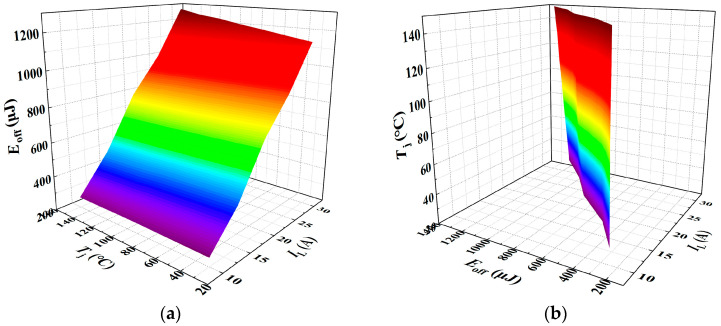
A 3D parametric model of *E*_off_ = *f*(*T*_j_, *I*_L_): (**a**) *E*_off_ as dependent variable in multivariate analysis; (**b**) *T*_j_ as dependent variable in multivariate analysis. The color gradient in the 3D plot primarily reflects variations in the vertical-axis amplitude, functioning as a contour-like representation. The transition from purple to red corresponds to changes in the amplitude value. This chromatic encoding visually enhances the interpretability of how the dependent variable evolves with the independent variables within the three-dimensional space.

Similarly, *E*_off_ was characterized under various junction temperatures and load currents, with the experimental results presented in Figure 17. Figure 17a,b, respectively, illustrate the variation of *E*_off_ with junction temperature under different load currents, and the variation of *E*_off_ with load current under different junction temperatures. The experimental results indicate that the current fall time during the turn-off process increases approximately linearly with both junction temperature and load current. Figure 18 presents the three-dimensional surface model of *E*_off_-*T*_j_-*I*_L_. When *E*_off_ is treated as the dependent variable, the fitted multiple linear regression model is presented in Equation (32), yielding a goodness of fit *R*^2^ of 99.43%. When *T*_j_ is considered the dependent variable, the corresponding regression model is shown in Equation (33), with a goodness of fit of 45.95%.

(32) $$E_{\mathrm{off}}=0.61383T_{j}+47.91057I_{L}-289.88571$$

(33) $$T_{j}=-38.34555I_{L}+0.80036E_{\mathrm{off}}+277.79661$$

The fitted multiple linear regression models of *t*_off_-*T*_j_-*I*_L_ and *E*_off_-*T*_j_-*I*_L_ indicate that both *t*_off_ and *E*_off_ exhibit an approximately linear increase with rising junction temperature. The regression models established with *t*_off_ and *E*_off_ as dependent variables and *T*_j_ and *I*_L_ as independent variables demonstrate a high goodness of fit, effectively capturing the variations in TSEPs induced by changes in junction temperature and load current. However, when *T*_j_ is treated as the dependent variable, the goodness of fit of the regression models is relatively low. Specifically, the goodness of fit *R*^2^ for the *T*_j_-*I*_L_-*t*_off_ model is 73.85%, while that for the *T*_j_-*I*_L_-*E*_off_ model is only 45.95%.

**Figure 19:**
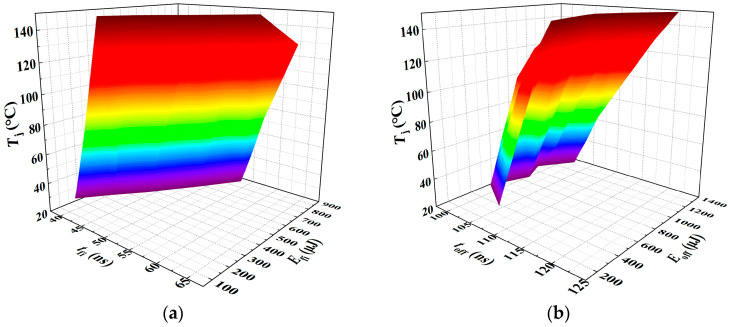
*T*_j_-*t*_fi_-*E*_fi_ and *T*_j_-*t*_off_-*E*_off_ three-dimensional models: (**a**) 3D parametric model of *T*_j_ = *f*(*t*_fi_, *E*_fi_); (**b**) 3D parametric model of *T*_j_ = *f*(*t*_off_, *E*_off_). The color gradient in the 3D plot primarily reflects variations in the vertical-axis amplitude, functioning as a contour-like representation. The transition from purple to red corresponds to changes in the amplitude value. This chromatic encoding visually enhances the interpretability of how the dependent variable evolves with the independent variables within the three-dimensional space.

Based on the fitting results of the junction temperature prediction models developed for the turn-off process and the current fall stage using TSEPs, it is evident that models relying on a single temperature-sensitive electrical parameter to back-calculate the junction temperature from operating conditions exhibit limited goodness of fit and poor prediction accuracy. To eliminate the influence of load current on junction temperature prediction, multiple TSEPs were coupled in the multiple linear regression model. Figure 19 presents the *T*_j_-*t*_fi_-*E*_fi_ and *T*_j_-*t*_off_-*E*_off_ three-dimensional models, respectively.

These models incorporate the TSEPs from the current fall stage and the turn-off process to construct and fit improved junction temperature prediction models. When junction temperature *T*_j_ is used as the dependent variable, and *t*_fi_ and *E*_fi_ are the independent variables, the resulting multiple linear regression model is presented in Equation (34), yielding a goodness of fit *R*^2^ of 94.11%. Similarly, when *T*_j_ is modeled as a function of *t*_off_ and *E*_off_, the corresponding regression model is given in Equation (35), with a goodness of fit *R*^2^ of 75.41%.

(34) $$T_{j}=16.96642t_{\mathrm{fi}}-0.43547E_{\mathrm{fi}}-605.07921$$

(35) $$T_{j}=7.23313t_{\mathrm{off}}-0.01057E_{\mathrm{off}}-694.1756$$

The goodness of fit comparison of the temperature prediction models established using a single temperature-sensitive electrical parameter and multiple linear regression based on coupled TSEPs is shown in Table 3.

As shown in the comparison results of the fitting accuracy of the three-dimensional model of TSEPs in Table 2, the proposed multiple linear regression method for junction temperature prediction based on the current fall phase characteristics of SiC MOSFETs demonstrates high accuracy, with an improvement of 5.23% compared to using the single temperature-sensitive electrical parameter *t*_fi_. Furthermore, coupling temperature-sensitive parameters from the turn-off process also enhances prediction accuracy relative to single-parameter models, thereby confirming the effectiveness and feasibility of the selected parameters. Additionally, comparison of the experimental results reveals that, for a junction temperature prediction model based on multiple TSEPs, its prediction accuracy depends on the combined contribution of each TSEP. The greater the temperature sensitivity of the individual TSEPs, the higher the prediction accuracy achieved by their coupled junction temperature model. Specifically, the *T*_j_-*t*_fi_-*E*_fi_ junction temperature prediction model established using coupled TSEPs from the current fall phase achieves higher accuracy than the *T*_j_-*t*_off_-*E*_off_ model based on the turn-off process, indicating its greater suitability for junction temperature estimation in SiC MOSFETs.

Table 4 presents a comparison of TSEPS for junction temperature measurement. Compared with other methods, the proposed approach exhibits better linearity and higher goodness of fit when applied to SiC MOSFETs. It requires no additional auxiliary circuitry, is well-suited for online monitoring, and does not interfere with the intrinsic characteristics of the circuit. Furthermore, the influence of load current is eliminated, thereby improving the accuracy of the temperature-sensitive electrical parameter-based junction temperature estimation model. These features not only validate the model’s estimation accuracy but also highlight its potential for integration into compact and low-cost embedded temperature-sensing systems for SiC-based power electronics.

## 5. Conclusions

This paper proposes an MLR method for junction temperature prediction based on the current fall characteristics of SiC MOSFETs. First, the feasibility of utilizing current fall time and current fall energy loss as TSEPs is demonstrated through theoretical analysis and simulation. Subsequently, an MLR junction temperature estimation model is constructed based on the three-dimensional mapping relationships between *t*_fi_-*T*_j_-*I*_L_, *E*_fi_-*T*_j_-*I*_L_, and *T*_j_-*t*_fi_-*E*_fi_. Experimental results demonstrate that coupling the two TSEPs, *t*_fi_ and *E*_fi_, effectively mitigates the influence of load current on model accuracy, thereby enhancing the precision of the MLR junction temperature prediction model. Compared to using *t*_fi_ as the sole temperature-sensitive electrical parameter, the model accuracy improved by 5.23%. Additionally, compared to the *T*_j_-*t*_off_-*E*_off_ prediction model based on turn-off process characteristics, the proposed *T*_j_-*t*_fi_-*E*_fi_ model demonstrates superior suitability for junction temperature estimation in SiC MOSFETs. This study not only validates the feasibility and effectiveness of the proposed approach but also demonstrates its potential for practical deployment in compact, low-cost junction temperature sensing systems. The model’s non-intrusive nature and high accuracy make it well-suited for real-time thermal monitoring, health management, and reliability assessment in SiC-based power electronic applications.

## Figures and Tables

**Table 1:** Parameters of the simulated SiC MOSFET circuit.

Parameter	Value
Bus Voltage *V*_DC_ (V)	600
Load Current *I*_L_ (A)	10–30
Load Inductance *L*_load_ (μH)	460
Gate Drive Voltage *V*_DRV_ (V)	+18/−3
External Gate Resistor *R*_G(ext)_ (Ω)	4.5
Internal Gate Resistor *R*_G(int)_ (Ω)	10.5
Loop Stray Resistance *R*_loop_ (Ω)	0.1
Source Parasitic Inductance *L*_S_ (nH)	10
Gate Parasitic Inductance *L*_G_ (nH)	15
Drain Parasitic Inductance *L*_D_ (nH)	20

**Table 2:** Parameters of the SiC MOSFET double-pulse test platform.

Parameter	Value
Bus Voltage *V*_DC_ (V)	600
Load Current *I*_L_ (A)	10–30
Load Inductance *L*_load_ (μH)	460
Gate Drive Voltage *V*_DRV_ (V)	+18/−3
External Gate Resistor *R*_G(ext)_ (Ω)	4.5
Internal Gate Resistor *R*_G(int)_ (Ω)	10.5

**Table 3:** Goodness of fit of each TSEP.

3D Model	Goodness of Fit *R*^2^
*t*_fi_-*T*_j_-*I*_L_	88.88%
*E*_fi_-*T*_j_-*I*_L_	28.91%
*T*_j_-*t*_fi_-*E*_fi_	94.11%
*t*_off_-*T*_j_-*I*_L_	73.53%
*E*_off_-*T*_j_-*I*_L_	45.95%
*T*_j_-*t*_off_-*E*_off_	75.41%

**Table 4:** Summary of TSEPS for junction temperature measurement.

TSEP	Ref.	Device	Linearity	Real-Time Implementation	Properties
Gate resistance	[18,21]	MOSFET/IGBT	Medium	Medium	Highly susceptible to noise.
Turn-on/off delay time	[19,25,26]	MOSFET/IGBT	Good	Hard	A large gate resistance needs to achieve detectable resolution.
Turn-on/off d*i*/d*t*	[20]	MOSFET	Low	Hard	Needs very high detection resolution and accuracy.
On-resistance	[22]	MOSFET	Medium	Easy	An additional high-precision drain-source voltage sensing circuit is required.
Gate turn-on peak current	[23,24]	MOSFET	Medium	Hard	Good for slow switching application.
Voltage rise time and voltage rise loss	[28]	IGBT	Medium	Medium	When applied to MOSFETs, the linearity is insufficient, resulting in a lower goodness of fit for the model.
The proposed method	/	MOSFET	Good	Medium	When applied to MOSFETs, the linearity is better, leading to a higher goodness of fit for the model.

## Data Availability

The original contributions presented in this study are included in this article; further inquiries can be directed to the corresponding author.
